# A-to-I RNA Editing Uncovers Hidden Signals of Adaptive Genome Evolution in Animals

**DOI:** 10.1093/gbe/evaa046

**Published:** 2020-03-07

**Authors:** Niko Popitsch, Christian D Huber, Ilana Buchumenski, Eli Eisenberg, Michael Jantsch, Arndt von Haeseler, Miguel Gallach

**Affiliations:** e1 Oxford NIHR Biomedical Research Center, Wellcome Trust Center for Human Genetics, University of Oxford, Oxford, United Kingdom; e2 Institute of Molecular Biotechnology (IMBA), Vienna BioCenter (VBC), Vienna, Austria; e3 Australian Centre for Ancient DNA, The University of Adelaide, Adelaide, South Australia, Australia; e4 The Mina and Everard Goodman Faculty of Life Sciences, Bar-Ilan University, Ramat Gan, Israel; e5 Raymond and Beverly Sackler School of Physics and Astronomy and Sagol School of Neuroscience, Tel Aviv University, Tel Aviv, Israel; e6 Department for Cell and Developmental Biology, Center for Anatomy and Cell Biology, Medical University of Vienna, Vienna, Austria; e7 Department for Medical Biochemistry, Max F. Perutz Laboratories, Medical University of Vienna, Vienna, Austria; e8 Bioinformatics and Computational Biology, Faculty of Computer Science, University of Vienna, Vienna, Austria; e9 Center for Integrative Bioinformatics Vienna, Max F. Perutz Laboratories, University of Vienna and Medical University of Vienna, Vienna, Austria; e10 iLabSystems, C/Alicante, Castellón, Spain

**Keywords:** RNA editing, human, *Drosophila*, natural selection

## Abstract

In animals, the most common type of RNA editing is the deamination of adenosines (A) into inosines (I). Because inosines basepair with cytosines (C), they are interpreted as guanosines (G) by the cellular machinery and genomically encoded G alleles at edited sites mimic the function of edited RNAs. The contribution of this hardwiring effect on genome evolution remains obscure. We looked for population genomics signatures of adaptive evolution associated with A-to-I RNA edited sites in humans and *Drosophila melanogaster*. We found that single nucleotide polymorphisms at edited sites occur 3 (humans) to 15 times (*Drosophila*) more often than at unedited sites, the nucleotide G is virtually the unique alternative allele at edited sites and G alleles segregate at higher frequency at edited sites than at unedited sites. Our study reveals that a significant fraction of coding synonymous and nonsynonymous as well as silent and intergenic A-to-I RNA editing sites are likely adaptive in the distantly related human and *Drosophila* lineages.

Through a single nucleotide modification, A-to-I RNA editing may impact the stability of the corresponding RNA molecule, recode the original protein sequence, and eventually modulate its biological function. The role of RNA editing in animal evolution is not well understood. A widely accepted hypothesis suggests that A-to-I RNA editing at nonsynonymous sites would entail a selective advantage over a genomic G nucleotide, as it increases the transcriptome diversity without affecting the genomically encoded A phenotype in tissues where editing does not occur (Gommans et al. 2009; Li et al. 2009; Nishikura 2010). This hypothesis predicts that edited A nucleotide sites will be rarely substituted by G nucleotides compared with unedited A sites (hypothesis H1, table 1). Contrary to this prediction, it was shown that A-to-G nucleotide substitutions between species are more frequent at edited sites than at unedited sites (Pinto et al. 2014; Xu and Zhang 2014). An alternative hypothesis (hypothesis H2, table 1) suggests that nonsynonymous A-to-G nucleotide substitutions between species are more tolerated (i.e., less deleterious) at edited sites than at unedited sites (Xu and Zhang 2014), explaining the difference in A-to-G substitution rates at nonsynonymous sites. Finally, a third hypothesis (hypothesis H3, table 1) proposes that G nucleotide sites are the ancestral state of currently edited A sites, and that A-to-I RNA editing is a compensation mechanism to reverse the harmful A phenotype caused by G-to-A mutations (Tian et al. 2008; Chen 2013; Pinto et al. 2014). However, the editing level, or fraction of edited molecules among the RNA copies of a given editing target, is far below 100%. For instance, in *Drosophila melanogaster*, the average editing level is 23% (St Laurent et al. 2013). This suggests that A-to-I RNA editing would rarely overcome the deleterious effects of the G-to-A mutations. In other words, in order to neutralize a deleterious G-to-A mutation in an organism, the A allele would need to be edited at a level closer to 100%. In addition, this hypothesis assumes that A-to-I editing would occur right after the derived A allele is originated, which is quite unlikely unless the G-to-A mutation occurs in the appropriate genomic sequence context (Zhang et al. 2017). In any case, each hypothesis predicts different evolutionary outcomes for the nonsynonymous edited sites compared with unedited sites (table 1) and points out our little understanding of this phenomenon.

**Table 1:** Hypotheses Suggested for the Evolution of A-to-I RNA Editing Target Sites

	Hypotheses			
	H1: Transcriptome Diversity Is Beneficial	H2: G Is Slightly Deleterious	H3: Compensatory Hypothesis	H4: Adaptive Hypothesis (Current Study)
Features				
Ancestral state	A	A	G	A
Adaptive value of editing	Editing is adaptive because provides diversity to transcript population.	Editing is very deleterious and currently detected edited sites are generally slightly deleterious.	Editing is adaptive as it reverses the harmful effect of G-to-A mutations.	Editing is adaptive because A-to-I replacements are beneficial at these nucleotide sites.
Relative fitness (*s*) of the derived allele	*s*_A_ > *s*_G_ ≥ *s*_C,T_	*s*_A_ ≥ *s*_G_ ≥ *s*_C, T_	*s*_G_ ≥ *s*_A_ > *s*_C,T_	*s*_G_ > *s*_A_ ≫ *s*_C,T_
Population genetics predictions compared with unedited sites				
Overall polymorphic rate	Polymorphism at edited sites should be *reduced* as A-to-G, A-to-C, and A-to-T mutations are slightly deleterious.	Polymorphism at edited sites should be *slightly increased* as A-to-G mutations are slightly more tolerated than at unedited sites.	Polymorphism at edited sites should be *similar or slightly increased* as editing somehow reduces the deleterious effect of G-to-A mutations.	Polymorphism at edited sites should be *increased* as A-to-G mutations are largely adaptive.
Polymorphism type	*A, G should be slightly more frequent* than A, C and A, T polymorphisms at edited sites.	*A, G should be slightly more frequent* than A, C and A, T polymorphisms at edited sites.	*A, G should be slightly more frequent* than A, C and A, T polymorphisms at edited sites.	*A, C and A, T polymorphism should be rarely found.*
Polymorphic rate at coding regions	*Similar or reduced* at both edited and unedited sites due to potential deleterious effects at nonsynonymous sites.	*Similar or reduced* at both edited and unedited sites due to potential deleterious effects at nonsynonymous sites.	*Similar or reduced* at both edited and unedited sites due to potential deleterious effects at nonsynonymous sites.	*Increased* at edited sites as the G allele mimics the protein variant obtained through editing.
Synonymous polymorphic rate	*Similar* at both edited and unedited sites.	*Similar* at both edited and unedited sites.	*Similar* at both edited and unedited sites.	*Increased* at edited sites.
Frequency spectrum of the derived allele	*Derived G* allele should segregate at *similar or lower* frequency (i.e., purifying selection or neutral at most).	*Derived G* allele should segregate at *similar* frequency (i.e., neutral or nearly neutral).	*Derived G* allele should segregate at *similar* frequency (i.e., neutral or nearly neutral).	*Derived G* allele should segregate at *higher* frequency.
Nucleotide diversity around edited sites	*Similar*	*Similar*	*Similar*	*Reduced*

To our knowledge, most studies have applied a phylogenetic approach to detect footprints of adaptive evolution of A-to-I RNA editing at coding regions (Xu and Zhang 2015; Yu et al. 2016; Duan et al. 2017; Zhang et al. 2017). Here, we employ a population genomics approach to search for signatures of selection in both coding and noncoding regions of the genome. To this end, we integrated the *D. melanogaster* and human editomes into population genomics data and investigated the population genetic patterns of the A-to-I RNA editing sites. Our study contradicts some predictions from previously suggested hypotheses and proposes a new adaptive role of A-to-I RNA editing in *Drosophila* and humans that may contribute to our understanding of the evolutionary patterns found at the editing sites.

## Results

### Differentiated Polymorphism Pattern at Edited and Unedited Sites in *Drosophila*

We analyzed *D. melanogaster* genome data from the *Drosophila* Genetics Reference Panel 2 (DGRP2) (Huang et al. 2014), consisting of 205 sequenced inbred lines derived from Raleigh (NC), United States, and two additional wild populations collected in Florida (FL) and Maine (ME), United States, consisting of 39 and 86 pool-sequenced inbred lines, respectively (Fabian et al. 2012). We investigated genome-wide nucleotide polymorphisms across more than 171 million nucleotide sites, 3,581 of them corresponding to known edited sites occurring in 1,074 genes (St Laurent et al. 2013). We found that 15% (FL and ME) to 21% (DGRP2) of the edited sites are polymorphic, in sharp contrast to the 1–2% found among genome-wide unedited sites (table 2). This result does not support hypothesis H1 (table 1), which predicts reduced polymorphisms at edited sites, but may be compatible with the hypotheses H2 and H3 (table 1) which predict similar or slightly increased polymorphism at coding edited sites. Thus, according to the original study from where hypothesis H2 is derived (Xu and Zhang 2014), A-to-G nonsynonymous substitutions at edited sites are twice as frequent compared with nonsynonymous unedited sites (6.92%/2.98%* *=* *2.32). Although this study (Xu and Zhang 2014) compares humans and mice (not *Drosophila*), the 2.32-fold difference is far below the 10- (DGRP2) to 15-fold (FL and ME) increase in polymorphic rate at edited sites. We did not find a clear quantitative prediction for hypothesis H3 (Tian et al. 2008; Chen 2013; Pinto et al. 2014). Remarkably, we found that the G nucleotide is the alternative allele in at least 98% of the polymorphic edited sites (including both silent and nonsynonymous ones), but only in ∼47% of the unedited polymorphic sites (table 2). The percentage of each polymorphism type at unedited sites fits the transition (A-to-G) and transversion mutation (A-to-C and A-to-T) frequencies in *Drosophila* (Keightley et al. 2009). This result seems incompatible with hypotheses H1–H3 as all polymorphism types should be found (table 1). Furthermore, when only considering introns, the G nucleotide is the alternative allele in 98.2% of the edited polymorphic sites (not shown). Thus, selection on coding sites (e.g., codon preference in *Drosophila*) does not explain the lack of C and T alleles at edited sites because this lack is also observed at edited sites in introns.

**Table 2:** Number of Single Nucleotide Polymorphism Sites and Polymorphism Types Among Edited and Unedited Sites in *Drosophila* Populations and Human

	DGRP2		FL		ME		Human–genic^c^		Human–intergenic^c^	
	Edited (%)	Unedited^b^ (%)	Edited (%)	Unedited (%)	Edited (%)	Unedited (%)	Edited (%)	Unedited (%)	Edited (%)	Unedited (%)
Polymorphic	**755 (21)**	3,951,070 (2)	**543 (15)**	1,367,160 (1)	**507 (14)**	1,235,454 (1)	**231 (19)**	176,080 (6)	**110 (18)**	196,030 (6)
Not polymorphic	2,826 (79)	171,048,930 (98)	3,038 (85)	118,920,513 (99)	3,074 (86)	119,052,219 (99)	977 (81)	2,811,804 (94)	520 (82)	3,017,246 (94)
Polymorphism^a^										
A, G	**740 (98)**	817,333 (45)	**536 (99)**	337,098 (48)	**502 (99)**	309,347 (49)	**225 (97)**	102,842 (58)	**105 (96)**	112,936 (58)
A, C	3 (0)	355,952 (20)	1 (0)	142,183 (21)	0 (0)	131,624 (20)	4 (2)	35,491 (21)	3 (3)	38,772 (20)
A, T	12 (2)	649,599 (35)	6 (1)	217,230 (31)	5 (1)	195,528 (31)	2 (1)	37,747 (21)	2 (1)	42,971 (22)

Note.—Bold values represent increased proportion in edited sites compared with unedited sites.

^a^ Only biallelic polymorphisms.

^b^ Assuming an average genome coverage of 175 Mb over the 205 lines (Huang et al. 2014).

^c^ Polarized data.

These observations hold two important implications: 1) because C and T alleles are virtually absent at edited sites, A-to-I RNA editing is functionally constrained and likely adaptive relative to C and T and 2) unless the A-to-G mutation rate is much higher at edited sites than at unedited sites due to an unknown molecular mechanism, the 10–15-fold increase in nucleotide polymorphism suggest that the G allele might be adaptive at edited sites relative to edited A (hypothesis H4, table 1). We thus looked for additional evidence supporting or disproving any of the four hypotheses.

### Differentiated Patterns Between Edited and Unedited Sites Persist at Silent Polymorphisms

Among the 3,581 edited sites in *Drosophila*, 1,015 are protein-coding nucleotides. Because of the potential deleterious effects caused by mutations in coding regions, nucleotide polymorphisms in such regions are expected to be similar or even lower than in noncoding regions (Begun et al. 2007). This is what we see for unedited sites, where nucleotide polymorphisms remain at 2% (DGRP2) or even decreases from 1% to 0.5% (FL and ME; supplementary table 1, Supplementary Material online). In contrast, nucleotide polymorphism at edited sites increases, on average, from 17% to 25% if we only consider coding regions. In other words, edited sites show a 16–44 times higher polymorphic rate than unedited sites at coding regions (supplementary table 1, Supplementary Material online).

This result prompted us to further investigate the relative contribution of nonsynonymous and synonymous replacements to nucleotide polymorphism at edited and unedited sites. Thus, to understand the A, G polymorphism on a genome wide scale, we scanned the reference genome for coding A sites, where a G mutation would result in a synonymous change (see Xu and Zhang 2014). We found *S *=* *777,461 A sites in the reference genome that would result in synonymous changes if replaced by G, 84,246 of which are actual synonymous A, G polymorphisms in the DGRP2 population, thus leading to a genomic rate of synonymous A, G polymorphisms *f*_s_^DGRP2^* *=* *84,246/*S *=* *0.108. Similarly, we computed for edited sites the rate of synonymous A, G polymorphisms (251) per potentially synonymous A, G site (*S*^edited^* *=* *370) as *f*_s_^edited,DGRP2^* *=* *251/*S*^edited^* *=* *0.678. For the FL and ME populations, we computed *f*_s_^edited,FL^* *=* *0.524, *f*_s_^FL^* *=* *0.029, and *f*_s_^edited,ME^* *=* *0.511, *f*_s_^ME^* *=* *0.027, respectively. Therefore, the rate of synonymous A, G polymorphisms for edited sites is 6–19 times higher than for unedited sites in *Drosophila* (table 3). This result is inconsistent with previously suggested hypotheses (table 1) that predict similar rates of synonymous polymorphism at edited and unedited sites, as the deleterious effects of A-to-G mutations (hypothesis H2) and G-to-A mutations (hypothesis H3) are supposed to occur only at nonsynonymous substitutions (see for instance figure 5 in Xu and Zhang 2014). Remarkably, for nonsynonymous sites, the differences between rates are even more pronounced: *f*_n_^edited,DGRP2^* *=* *0.105 and *f*_n_^DGRP2^* *=* *0.007, which implies a 15-fold increased rate for edited nonsynonymous sites in DGRP2, whereas for the ME and FL populations, the rate increase is 45-fold and 51-fold, respectively (table 3).

**Table 3:** Potential A, G Synonymous and Nonsynonymous Replacements in *Drosophila* Populations

Population	Potential A, G Synonymous Replacements					Potential A, G Nonsynonymous Replacements				
	Edited (*S*^edited^* *=* *370)		Genome (*S *=* *777,461)		Ratio	Edited (*N*^edited^* *=* *645)		Genome (*N *=* *4,448,133)		Ratio
	Polymorphic	Rate (*f*_s_^edited^)	Polymorphic	Rate (*f*_s_)	*f*_s_^edited^/*f*_s_	Polymorphic	Rate (*f*_n_^edited^)	Polymorphic	Rate (*f*_n_)	*f*_n_^edited^/*f*_n_
DGRP2	251	0.678	84,246	0.108	6	68	0.105	29,727	0.007	15
ME	181	0.511	21,198	0.027	19	29	0.045	4,349	0.001	45
FL	194	0.524	22,603	0.029	18	33	0.051	4,647	0.001	51

A common way to determine the evolutionary force driving coding sequence evolution is the ratio of the number of nonsynonymous substitutions per nonsynonymous site (*d*_N_) to the number of synonymous substitutions per synonymous site (*d*_S_). The estimates of *f*_s_ and *f*_n_ fall within the distribution of *d*_S_ (0.030–0.128; 5th and 95th percentiles, respectively) and *d*_N_ (0.000–0.022; minimum and 95th percentile, respectively) estimations for *D. melanogaster* genes (Singh et al. 2008). We therefore applied the same reasoning behind the *d*_N_/*d*_S_ ratio (Hurst 2002) to our *f*_s_ and *f*_n_ estimations. This is: If selection does not act on synonymous sites, then *f*_n_^edited^/*f*_s_^edited^ > 1 may be considered as an evidence of positive selection on nonsynonymous edited sites (Akashi et al. 2012; Xu and Zhang 2014). However, the large polymorphism rate that we observe for edited sites and the fact that *f*_s_^edited(mean)^* *∼* *14* *×* f*_s_^mean^ indicates that edited synonymous sites are not neutral but likely adaptive (see also Derived G Alleles at Edited Sites Are Likely Adaptive in Drosophila) due to the pervasive roles of RNA editing in the posttranscriptional regulation of gene expression (Chamary et al. 2006; Wang et al. 2013). We therefore used *f*_s_^mean^* *=* *0.055 as the neutral rate for synonymous A, G polymorphisms in the genome, and obtained *f*_n_^edited(mean)^/*f*_s_^mean^* *=* *1.34 (*P *=* *0.012, one-sided Binomial test for the null hypothesis *f*_n_^edited(mean)^* *≤* f*_s_^mean^). We get a similar ratio >1 when comparing the polymorphism rate of nonsynonymous edited sites with intronic sites (*f*_n_^edited(mean)^/*f*_intron_^mean^* *=* *2.01), suggesting that selection at synonymous sites cannot explain this ratio. We conclude that, overall, the alleles encoding the same protein variant that is obtained through A-to-I RNA editing are likely adaptive.

### Derived G Alleles at Edited Sites Are Likely Adaptive in *Drosophila*

**Fig. 1.—:**
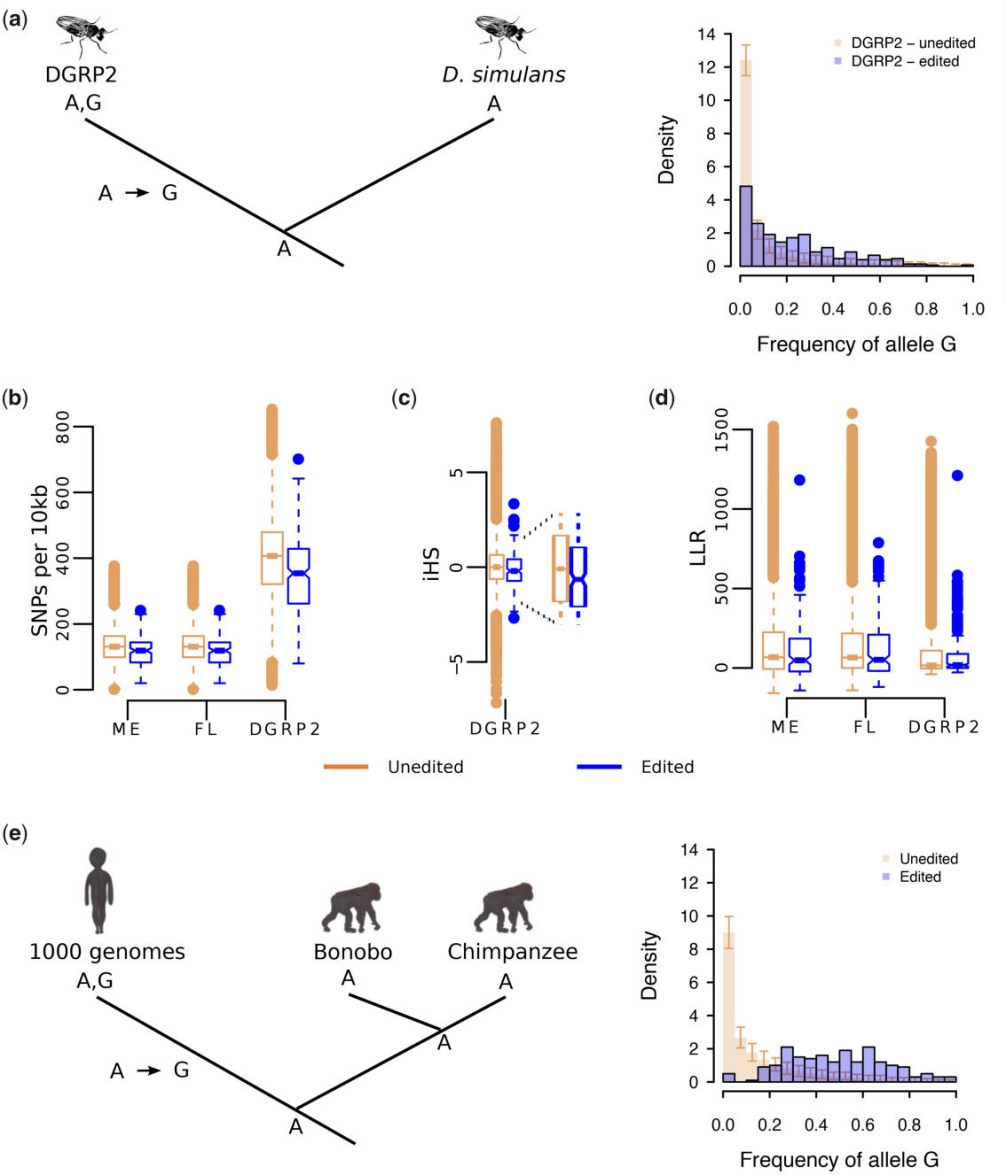
Properties of the G alleles segregating at edited sites in *Drosophila melanogaster* and human. (*a*) We used *D. simulans* as an outgroup to infer the ancestral state of the A, G polymorphisms in *D. melanogaster*. The right panel shows the average frequency spectrum and 95% confidence interval of the derived G alleles at unedited sites (peach) and the frequency spectrum for the derived G alleles at edited sites (blue). The shift of the blue distribution toward higher G allele frequencies is a signal of positive selection for the derived G alleles at edited sites. The frequency shows an average increase of 0.12. (*b*) Windows centered on polarized A-to-G mutations have lower diversity (in SNPs per 10 kb) for edited SNPs than for unedited SNPs (*P *<* *10^−4^ for each paired comparison; one-sided Wilcoxon rank sum test). (*c*) At polarized edited sites, the extended homozygosity of the haplotype carrying the derived G allele is longer than that of the haplotypes carrying the ancestral A allele (average iHS score < 0). At unedited sites, the extended homozygosity is similar for both haplotypes (average iHS score ∼ 0). *P *=* *0.004, one-sided Wilcoxon rank sum test for the null hypothesis iHS (edited) ≥ iHS (unedited). (*d*) The LLR comparing a long-term balancing selection model versus a neutral model tend to be lower for edited sites than for unedited sites (expected to be higher if balancing selection were more prominent for edited sites). *P *≫* *0.05 for each paired comparison; two-sided Wilcoxon rank sum test. (*e*) We used Bonobo and Chimpanzee as an outgroup to infer the ancestral state of the genic A, G polymorphisms in the human genome. The right panel shows that G alleles segregate at higher frequencies in edited sites (blue) than in unedited sites (peach). The frequency shows an average increase of 0.34.

According to population genetics theory, if the G alleles at polymorphic edited sites were adaptive, they would segregate at higher frequencies than G alleles at unedited sites originated at the same time (Kimura 1983). This effect should be detectable by comparing the allele frequency spectrum for edited and unedited A, G polymorphisms. We used *D. simulans* population genomics data (Begun et al. 2007) to infer the ancestral state (i.e., polarize) of the polymorphic A sites across the genome in the DGRP2 population and to be confident that the derived G alleles at edited and unedited sites are of similar age. We detected 462,498 A-to-G polymorphisms across the genome, where the (derived) G allele most likely originated in *D. melanogaster’*s lineage, 303 of them occurring at edited sites (supplementary table 2, Supplementary Material online). Figure 1*a* displays the allele frequency spectrum of the derived G alleles at edited and unedited A-to-G polymorphic sites. Remarkably, the frequency spectrum for the derived G alleles at edited sites is shifted to the right and quite distinct from that of unedited sites, indicating that a significant fraction of A-to-G mutations at edited sites is likely adaptive. Our analysis in FL and ME populations supports this observation (supplementary figs. 1 and 2, Supplementary Material online). To control for local genomic properties that might differ between edited and unedited sites and might also cause the shift in frequency we have generated a control set of unedited A-to-G sites. For each edited A-to-G site, we select the closest unedited A-to-G site. On average, these unedited control sites are only 132 bp away from the respective edited sites. In line with the previous analyses, we observe a significantly larger allele frequency of the G allele at the edited sites compared with the control sites (Wilcoxon rank sum test, *P* < 2.2e−16).

Furthermore, 266 (i.e., 88%) of the 303 polarized polymorphisms correspond to noncoding edited sites. When filtering for noncoding sites, we again find a significantly larger allele frequency of the G allele at edited compared with unedited sites (Wilcoxon rank sum test, *P* < 2.2e−16), revealing a likely functional role of noncoding edited sites. This result is incompatible with the hypotheses H2 and H3, as the frequency spectrum for the derived G-allele at noncoding edited sites should fit the neutral expectation and thus be the same as at noncoding unedited sites (table 1).

We repeated our analysis for polarized G-to-A mutations. If editing overcame the deleterious effects of G-to-A mutations (i.e., edited A allele becomes neutral or nearly neutral relative to the original G allele; hypothesis H3), the derived A alleles at edited sites would segregate at similar frequencies as derived A alleles at unedited sites. However, we found that derived A alleles at edited sites segregate at a remarkable lower frequency than derived A alleles at unedited sites (supplementary fig. 3a, Supplementary Material online). This result seems incompatible with hypothesis H3, especially if we consider silent sites (supplementary fig. 3b, Supplementary Material online) but it fits the prediction from hypothesis H4: Because edited A alleles are less preferred than G alleles, edited A sites will segregate at lower frequency.

### Differentiated Genomic Footprints Around Edited Sites in *Drosophila*

Mutational bias at edited sites, if it existed, would not explain the different allele frequency distribution between edited and unedited sites. Only two different scenarios may explain the higher frequency of the derived G allele at edited sites: directional selection in favor of the G allele or long-term balancing selection. Thus, we further looked for genomic signatures across the polarized A-to-G polymorphisms that helped us to distinguish between these two scenarios.

According to the theory of selective sweeps, a new adaptive mutation appears on a single haplotype that quickly goes to fixation due to directional selection. The hallmark of a selective sweep is a reduction of nucleotide diversity near the adaptive mutation (Olson-Manning et al. 2012). Accordingly, if the G allele at edited sites is positively selected, we expect reduced nucleotide diversity in genomic regions around polymorphic edited sites compared with unedited sites. We computed the number of single nucleotide polymorphisms (SNPs) in 10 kb windows centered on edited A-to-G polymorphisms across the genome and tested whether these windows had the same nucleotide diversity than those centered on unedited A-to-G polymorphisms (fig. 1*b*). The average number of SNPs are 346, 125, and 116 for windows centered on edited sites (DGRP, FL, and ME, respectively) and 398, 144, and 131 for windows centered on unedited sites (DGRP, FL, and ME, respectively). Such a reduction of nucleotide diversity is significant in the three populations (*P *<* *10^−4^ for each paired comparison; one-sided Mann–Whitney *U* test) and a similar reduction of diversity is observed for 1 kb windows (supplementary fig. 4, Supplementary Material online).

Further, we tested if the addition of recombination rate and GC content as covariates in an analysis of covariance analysis can explain the effect of editing on diversity. The dependent variable is polymorphism divided by divergence to *D. simulans* in 10 kb windows, that is, controlling for mutation rate and purifying selection similar to an Hudson–Kreitman–Aguadé (HKA) test. We find that both recombination rate and GC content have a significant effect on the polymorphism over divergence ratio, however, they do not remove the significant effect of editing. The effect of editing becomes even stronger when filtering for larger allele frequencies at the central site, which is compatible with a model of positive selection, where the effect of reduced diversity is stronger when the haplotype linked to the beneficial mutation has reached a higher frequency (supplementary table 5, Supplementary Material online). Thus, positive selection but not genomic confounding factors such as recombination rate, GC content, or purifying selection, can explain the reduced diversity around polymorphic edited sites.

Another prediction of directional selection is that, because the adaptive G allele increases in frequency relatively fast, it will locate on an unusually long haplotype of low nucleotide diversity (Voight et al. 2006). On the other hand, the haplotypes carrying the original A allele should be shorter than the haplotypes carrying the adaptive G allele but of similar length to haplotypes from a neutral genomic background. We used the genotypes of the 205 inbred lines from the DGRP2 to compute the integrated haplotype score (iHS) (Voight et al. 2006), an index that compares the extended homozygosity of the haplotypes carrying the derived G allele with that of the ancestral A allele. The iHS values at unedited A-to-G polymorphism (median iHS = 0.003) indicate that the haplotypes carrying the alleles at unedited SNPs have the same length and are likely neutral (Voight et al. 2006). In contrast, the negative median iHS = −0.202 at edited A-to-G polymorphism (fig. 1*c*) indicate unusually long haplotypes carrying the derived G allele and suggest that these haplotypes have increased in frequency faster than neutral expectation. This difference in the overall distribution of the iHS statistic is significant (*P* = 0.004, one-sided Wilcoxon rank sum test for the null hypothesis iHS (edited) ≥ iHS(unedited)). However, when testing individual edited site at a time, only 12 of the iHS values are significant (*P *<* *0.05, one-sided *t* test for the null hypothesis iHS^edited^ ≤ iHS^unedited^), revealing some limitations of our analysis (see Discussion for further details). Further, we also tested if recombination rate and GC content can explain the effect of editing on the iHS distribution (supplementary table 5, Supplementary Material online). We find that when filtering for sites with large G frequency at the central site (>10%), editing has a significant effect on iHS. However, without filtering the effect becomes nonsignificant, suggesting that filtering helps to enrich for positively selected variants that have reached high frequencies, which increases power for haplotype methods such as iHS (Voight et al. 2006).

The reduced nucleotide diversity near the edited A-to-G polymorphism and the longer haplotypes carrying the derived G alleles at edited sites is inconsistent with long-term balancing selection, as a prediction of balancing selection is a local increase in nucleotide diversity (DeGiorgio et al. 2014). To further evaluate long-term balancing selection as one reason for the higher population frequency of the derived G allele at edited sites, we tested whether the local increase in nucleotide diversity relative to nucleotide divergence (i.e., fixed differences between species) is stronger near polymorphic edited sites than near polymorphic unedited sites (DeGiorgio et al. 2014). This likelihood ratio test, implemented in the software *BALLET*, is similar to the HKA approach but tests an explicit alternative model of balancing selection based on predictions from coalescent theory. We gathered a total of 100 nucleotide sites upstream and downstream of the polarized A-to-G polymorphisms across the genome, where a site is either an SNP or a fixed difference between *D. melanogaster* and *D. simulans*. For each window, we computed a log-likelihood ratio (LLR) that compares a balancing selection model against a neutral model based on the background genome pattern of polymorphisms (DeGiorgio et al. 2014). Our analysis shows that the likelihood of the balancing selection model relative to that of the neutral model is lower in windows centered on A-to-G polymorphic edited sites than in windows centered on A-to-G polymorphic unedited sites (fig. 1*d*). The average LLRs comparing both models are 78, 120, and 111 for windows centered on A-to-G edited sites (DGRP2, FL, and ME, respectively) and 83 and 136 for windows centered on A-to-G unedited sites (DGRP2 and both FL and ME, respectively). This result indicates that there is no effect of balancing selection at edited A-to-G sites.

### Differentiated Polymorphism Pattern and Allele Frequency Spectrum Between Edited and Unedited Sites of *Alu* Repeats

We further applied our comparative analysis in humans to determine whether the selective footprints found in *Drosophila* were unique to this lineage or, otherwise common between these two distantly related species. Because the human genome is about two orders of magnitude larger than *Drosophila*’s, several difficulties arose, in particular: The list of (coding) edited sites is proportionally shorter than in *Drosophila* (in part due to the filtering by SNPs that is normally done to annotate the human editome) and the proportion of homologous nucleotide sites sequenced in other apes’ genomes (needed to polarize polymorphisms) is greatly reduced. Consequently, our approach in humans is inevitably more challenging and limited than in *Drosophila*. For instance, in our first attempt to apply our approach to humans, we integrated a recent list of 2,042 known coding edited sites (Xu and Zhang 2015) into a population genomics database compiled from the 1,000 Genomes Project (McVean et al. 2012) and the Great Ape Genome Project (Prado-Martinez et al. 2013). However, only 10 of the 2,042 edited sites were represented in our database, impeding any further genome-wide analysis.

Because humans have more than a million copies of *Alu* (Lander et al. 2001) and virtually all adenosines within *Alu* repeats that form double-stranded RNA undergo A-to-I editing (Bazak et al. 2014), we used our population genomic approach on *Alu*s. By using *Alu*s, we are limiting our analysis to silent (most genic *Alu* repeats occur in introns and 3′ UTRs) and intergenic A sites, but we gain in numbers enough to look for genome-wide polymorphism patterns. With this in mind, we analyzed RNA-Seq data from 105 control (healthy) breast samples from The Cancer Genome Atlas (TCGA) and annotated de novo a list of 28,322 highly edited sites at *Alu* repeats, 1,838 of them represented in our database (1,208 genic and 630 intergenic; table 2). Remarkably, we found a 3-fold increase in the nucleotide polymorphism at edited *Alu* sites (19%) compared with unedited *Alu* A sites (6%) located in genes. In addition, the G nucleotide is the alternative allele in 97% of the polymorphic edited sites, but only in 58% of the unedited polymorphic sites (table 2). We used chimpanzee and bonobo population genomic data to infer the ancestral state of the A, G polymorphisms occurring at genic *Alu*s and compared the frequency spectrum of the derived G alleles segregating at edited and unedited sites. Figure 1*e* shows that derived G alleles at edited sites segregate at higher frequency than derived G alleles at unedited sites. Notably, we observed a similar nucleotide polymorphism pattern (table 2) and allele frequency spectrum (supplementary fig. 6, Supplementary Material online) for edited sites in intergenic *Alu* repeats. Our study in humans therefore confirms our results in *Drosophila* and suggests that a significant fraction of A-to-G mutations at edited sites is also adaptive in humans, including those occurring in intergenic regions.

## Discussion

**Fig. 2.—:**
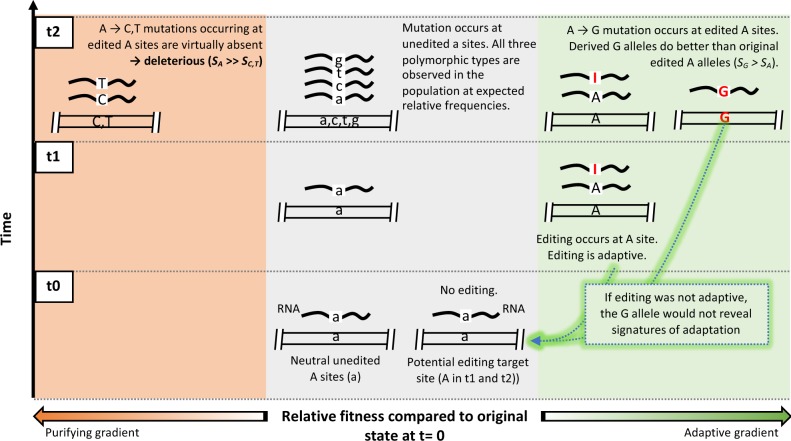
Illustration of an adaptive model for A-to-I RNA editing evolution. Our model suggests that the genomically encoded G nucleotide is generally adaptive at edited sites compared with the original edited A site because it mimics the function of the edited RNA (t2, right panel). This implies that A-to-I RNA editing is also adaptive (t1, right panel); otherwise, the G allele would not reveal signatures of positive selection (green arrows pointing to t0 at middle panel). If editing was neutral or deleterious, we would observe C and T alleles (t2, left panel), at least at silent sites (hypothesis H2). If G was the ancestral allele and A was detrimental, other detrimental alleles (C and T alleles, t2, left panel) would be observed as well, at least at A sites where editing level is low (hypothesis H3). Gray bars: genomic loci. Black lines: RNA molecules transcribed from the corresponding genomic loci. S: selection coefficient. A: edited A site; a: unedited A site; C, T, and G: derived alleles from edited A sites; c, t, and g: derived alleles from unedited A sites.

The binary classification (edited/unedited) of *Drosophila* and human population genomic data based on a posttranscriptional modification uncovered a selective footprint that, otherwise, would remain hidden. Some of these footprints seem incompatible with the current hypotheses on the evolution of A-to-I RNA editing and prompt us to suggest an additional hypothesis that may better explain our results and complement previous hypotheses (fig. 2).

The extraordinary differences of the polymorphic rates and polymorphism types between edited and unedited sites are very unlikely affected by differences in the usage of synonymous codons (supplementary fig. 7a, Supplementary Material online), gene expression level (supplementary fig. 7b, Supplementary Material online) or recombination rates (supplementary figs. 5 and 7c and supplementary table 5, Supplementary Material online) between edited and unedited sites. Higher GC-biased gene conversion (i.e., the unequal exchange of genetic material between homologous loci) is also an unlikely source of bias as there is no GC-biased gene conversion in *Drosophila* (Robinson et al. 2014) and we restricted our analysis in human to A sites of *Alu* elements, ensuring identical local sequence for both edited and unedited sites. In addition, we did not find significant differences in the nucleotide composition around edited and unedited A sites in *D. melanogaster* that might suggest context-driven local mutation rates (supplementary fig. 7d, Supplementary Material online). Yet, even if it existed, a biased A-to-G mutational rate at edited sites would not explain adaptive signatures at edited sites such as lower surrounding nucleotide diversity or the higher frequency of the derived G allele. Finally, we found similar results for *Drosophila* and human out of different editing annotation strategies and population genomic data sets, suggesting that annotation artifacts are not likely affecting our analysis. To positively discard this option, we have also confirmed the described patterns in other three available *Drosophila* editomes (supplementary fig. 8 and supplementary table 3, Supplementary Material online). Further, similar to mutational biases, the characteristic signatures of positive selection at linked neutral sites that we observe, and the absence of C and T alleles, are not predicted to result from false-positive calls of RNA editing and are thus robust to this potential error.

The fact that the nucleotides C and T are virtually absent at edited sites suggest strong functional constraints upon edited A sites in humans and flies. This implies that the relative fitness (*s*) of edited A sites is much higher than that of the alternative C and T alleles (*s*_A_ ≫ *s*_C, T_). In addition, the fact that derived G alleles at edited A sites segregate at higher frequencies than expected (fig. 1*a* and *e*) indicates that the A-to-G mutations at edited sites are generally adaptive. In other words: *s*_G_ > *s*_A_ ≫ *s*_C, T_ at edited sites. Because *s*_A_ < *s*_G_ at edited sites, derived A alleles (including silent polymorphisms) are not neutral nor nearly neutral even when edited (supplementary fig. 3a and b, Supplementary Material online). These observations are also difficult to explain according to the current hypotheses on editing (hypotheses H1–H3, table 1) and shed light on the adaptive roles of the G mutations at edited sites and on the A-to-I RNA editing itself. Our hypothesis is that a genomically encoded G nucleotide is generally adaptive at edited sites because it mimics the function of the edited RNA (fig. 2). In other words, encoded G nucleotide would be equivalent to a dominant edited A phenotype. This implies that A-to-I RNA editing is also generally adaptive (hypothesis H4, table 1 and fig. 2). If A-to-I RNA editing were not adaptive, the G allele would not reveal signatures of adaptation and C and T alleles would be also found at edited SNPs (both coding and noncoding).

We showed that directional selection in favor of the derived G allele is more likely than balancing selection acting at A, G polymorphic edited sites. However, the evidence is weak for several reasons. First, we can only analyze incomplete selective sweeps because we do not know which G nucleotide sites currently fixed in *D. melanogaster* were edited A sites in the past. Second, the selection strength may depend on the dominance of the derived G allele. For instance, it is likely that the dominance has a more prominent effect at nonsynonymous G mutations than at silent mutations. Third, although directional selection may be more prominent, balancing selection may still occur at some edited sites. Despite these limitations, by averaging over many sites, the footprint for directional selection, and not balancing selection, seems more evident but not conclusive. Weak positive selection of the G allele at many edited A sites may also explain the increased level of diversity at those sites as well as the shift in the site frequency spectrum to larger frequencies. However, differentiating between strong and positive selection of the derived G allele is difficult as derived G alleles at high frequencies will be found more often in homozygosis in inbred lines than low frequency ones. In consequence, we expect that polymorphic edited A sites whose derived G alleles segregate at low frequencies will be easier to annotate, favoring the weak positive selection hypothesis against the strong positive selection one.

The adaptive potential of A-to-I RNA editing by modifying the protein sequence have been recently proven. Garrett and Rosenthal (2012) showed that the editing level of the mRNA encoding the octopus’ potassium Kv1 channels correlates with the water temperature, where the octopus’ species were captured. Most importantly, a concomitant physiological amelioration at cold Antarctic temperatures indicates that RNA editing may play a significant role in thermal adaptation in this species. The important role of A-to-I RNA editing on posttranscriptional regulation, including editing of genic *Alu* sequences (Nishikura 2010), also suggest an adaptive potential of editing as a checkpoint to gene expression control. In summary, the adaptive role of the G mutation at edited sites may come in two ways: by encoding the same protein variant and “encoding” the same RNA secondary structure as in the edited RNA.

The adaptive role of the G mutations at edited A sites of intergenic *Alu* repeats is less obvious to explain. It has been shown that ADAR1 mutants overexpress genes containing edited *Alu* repeats and that *Alu* editing is involved in the nuclear retention of the cognate mRNA (Osenberg et al. 2010). We suggest that A-to-I RNA editing (and A-to-G mutations mimicking the editing function) might be an adaptive mechanism to prevent the deleterious effect of retrotransposition of intergenic *Alu* repeats by disrupting functional dsRNA structures of the repeats. By disrupting the functional dsRNA structure, the cell may: 1) silence the expression of the *Alu* repeats or 2) retain the transcribed *Alu* repeats to impede their retrotranscription in the cytoplasm. Because an encoded G nucleotide would mimic the edited isoform and disrupt the dsRNA structure as well, the nucleotide G would be preferred overedited A, as in the former case, all molecules would be disrupted.

We found surprisingly similar signatures of adaptation at edited sites in two distantly related species, humans and *D. melanogaster*. A careful consideration of the heterogeneous genomic confounding factors in humans is warranted in future studies. Nonetheless, we expect that new population genomics data and new editome annotations will help us to find additional signs of positive selection in other animal classes and confirm the pervasive adaptive potential that A-to-I RNA editing offers. Our novel approach will hopefully help to expose similar genome-wide adaptive patterns associated with the expanding epitranscriptome landscape.

## Methods

### Population Genomic Data

We downloaded the genotypes of the 205 inbred lines annotated in the DGRP2 (Huang et al. 2014) (http://dgrp2.gnets.ncsu.edu/). In addition, we also analyzed pooled DNA-Seq data from *D. melanogaster* flies collected in 2010 from outbred populations in ME (86 lines) and FL (39 lines) (Fabian et al. 2012). We trimmed 101 bp paired-end reads with ConDeTri (Smeds and Künstner 2011) using the following parameters: hq = 20, lq = 10, frac = 0.8, minlen = 50, mh = 5, ml = 1, and mapped with NextGenMap (Sedlazeck et al. 2013) the remaining reads longer than 50 bp to the *D. melanogaster* reference genome, release r5.40 (ftp://ftp.flybase.net/genomes/). Next, we removed reads with a mapping quality value lower than 20 with SAMtools (Li, 1000 Genome Project Data Processing Subgroup, et al. 2009). We called SNPs for each data set when the coverage was ≥10 at this nucleotide site and at least two reads carried the alternative allele.

A pileup from six *D. simulans*’ sequenced genomes was downloaded from the *Drosophila* Population Genomics Project (http://www.dpgp.org/). We used UCSC’s liftover tool (Hinrichs et al. 2006) to convert dm2 coordinates into dm3 coordinates (BDGP Release 5).

Primate population genomic data were downloaded from the Great Ape Genome Project (Prado-Martinez et al. 2013). We converted the coordinates from hg18 to hg19 using liftover and used hg19 nucleotide site ID to merge the Great Ape population genomics data with the human data from the 1,000 Genomes Project (McVean et al. 2012). The merged population genomics database consists of 179,546,112 entries indicating homologous nucleotide sites in great apes and allele frequency information in humans.

### A-to-I RNA Editing Data

An important caveat in editome annotation pipelines is filtering out SNP, which biases the A-to-I RNA editing list against standing genetic variation. In our study, we integrated databases avoiding this bias. In the main text, we used one of the most cited studies about the annotation of the A-to-I RNA editing sites in *D. melanogaster*, which consists of 3,581 sites (St Laurent et al. 2013). In this study, editing events were called when G allele expression was detected from a homozygous AA genotype. The potential editing sites were further confirmed by the absence of G allele expression at putative editing sites in ADAR^−/−^ mutants. In addition to this, we further integrated a list of 2,197 validated (Sanger sequencing) sites in St Laurent et al. (2013), a list of 5,025 editing sites from RADAR (a Rigorously Annotated Database of A-to-I RNA Editing) (Ramaswami and Li 2014), and a list of 1,299 editing sites from Yu et al. (2016).

We annotated de novo the A-to-I RNA editing sites occurring in *Alu* repeats in a conservative way. Briefly, we mapped RNA-Seq data from 105 control (healthy) breast tissue samples available at TCGA project (http://cancergenome.nih.gov/) against the human reference genome (hg19) with STAR aligner v2.3.0 (Dobin et al. 2013). Only uniquely mapped reads with <5% mismatches were kept for further analysis, allowing us to test a total of 148,961,882 A sites for A-to-I RNA editing. For the purpose of this study, we defined a site to be edited if 1) the G allele were found at >1% of the reads in >50% of the breast samples and 2) the G allele was not found in the dbSNP (build 146) at frequency >0.5. Otherwise, the A site was defined as unedited. This definition allowed us to detect 28,322 highly edited sites out of the ∼149 million A sites tested.

### Polarizing A-to-G Mutations in *D. melanogaster* and Human

We downloaded pairwise *D. melanogaster*/*D. simulans* axt alignment files from UCSC (http://hgdownload.soe.ucsc.edu/goldenPath/dm3/vsDroSim1/). A script was generated to parse the alignment files and detect the homologous sites in *D. simulans* reference genome and in six additional *D. simulans* genomes downloaded from the *Drosophila* Population Genomics Project (http://www.dpgp.org/).

We need to take into account how editing is annotated in *Drosophila* to determine the set of nucleotide sites to be polarized. The idea in editome annotation is to find A, G expression from a homozygous AA genotype. If the tested A nucleotide is fixed in the population, there are no sampling bias as all lines will be homozygous AA. However, if a site is polymorphic (e.g., it segregates A and G nucleotides), you need to sample an AA homozygous site (from the population or through inbreeding) to test for editing, and it will be easier to sample homozygous AA sites when A is the major allele than when A is the minor allele. This is why all polymorphic edited A, G sites have A as reference nucleotide, whereas unedited A, G polymorphism may have A or G as reference nucleotide. Therefore, A-to-G mutations were inferred to occur on the *D. melanogaster* lineage (DGRP, ME, and FL populations) when the homologous site in the *D. simulans* lines was A (i.e., monomorphic in *D. simulans* population), and for our analysis, we only consider those A-to-G mutations whose reference nucleotide is A in *D. melanogaster*.

The effect of the reference genome is easier to see when we polarize G-to-A mutations (compare supplementary fig. 3a with supplementary fig. 3c, Supplementary Material online). Because there is no edited G-to-A site whose reference nucleotide is G (all such sites have A as reference nucleotide), we only consider for our analysis polarized G-to-A sites whose reference nucleotide is A (supplementary fig. 3a and b, Supplementary Material online).

For our analysis in human, we parsed the pileup file from the Great Ape Genome Project and compiled the list of human A, G SNPs that likely originated by A-to-G mutation in the human lineage. The ancestral state of an A, G polymorphism was already inferred in the original study and stored in the pileup file as *node 18* (Prado-Martinez et al. 2013).

### Allele Frequency Spectrum

Low coverage in pool-sequencing experiments may inflate the frequency estimation of alleles segregating at low frequencies. We tested for different coverages among edited and unedited polymorphisms and for a correlation between coverage and minor allele frequency in ME and FL populations. Supplementary figure 2, Supplementary Material online, shows that the coverage is not different between edited and unedited sites and that allele frequency and coverage do not correlate. Therefore, we are confident that the higher frequency of the G allele in edited sites is not due to an artifact associated with coverage.

After polarizing the polymorphism data with *D. simulans*, we found 462,801; 110,844; and 125,807 A-to-G polymorphic sites in DGRP2, ME, and FL populations, respectively, that most likely originated from A-to-G mutations. 303, 155, and 179 of these sites are edited sites in DGRP2, ME, and FL populations, respectively (supplementary table 2, Supplementary Material online).

For DGRP2 data, we computed the frequency of the derived G allele as *π*_G_^DGRP2^ = GT_G_/(GT_G_ + GT_A_), where GT_G_ and GT_A_ are the number of lines with genotype GG and genotype AA, respectively. For ME and FL populations, we computed the frequency of the G allele as *π*_G_^ME, FL^ = *g*/*r*, as suggested for pool-sequencing data (Gautier et al. 2013), where *g* is the number of DNA-Seq reads carrying the G allele and *r* is the total number of reads mapped at this site. To compute the allele frequency spectrum of the derived G alleles across the genome, we sampled 303, 155, and 179 sites from the 462,801; 110,844; and 125,807 polarized A-to-G polymorphic sites in DGRP2, ME, and FL populations, respectively. We repeated the sampling 100,000 times (per population) to compute the average distribution and the 95% confidence interval for each frequency class.

We polarized 176,311 tested A, G human polymorphisms occurring at genes that most likely originated from A-to-G mutations; 231 of them corresponded to edited sites in genes (table 2). To compute the allele frequency spectrum of the G allele at genes, we sampled 231 sites from the 176,311 unedited A, G polymorphisms. We repeated the sampling 100,000 times and compute the average allele frequency spectrum and the 95% confidence interval for each frequency class. We took the frequency of the G alleles from the 1,000 Genomes Project. With regards to intergenic regions, we polarized 196,140 tested A, G human polymorphisms that most likely originated from A-to-G mutations; 110 of them corresponded to edited sites (table 2). The sampling procedure was as explained for genic A, G polymorphism with sampling size 110.

### Testing for Balancing Selection and Directional Selection

To test for directional selection in favor of the derived G allele in edited sites, we first tested whether diversity was lower around edited sites than around unedited sites. To this aim, we counted the number of SNPs in windows of 10 kb centered on each polarized A-to-G polymorphism. The ancestral allele was again determined based on data from *D. simulans*. We also used the recombination rate data from Comeron et al. (2012) to linearly interpolate local recombination for the 10 kb windows. The distribution of local recombination rates at edited and unedited sites are essentially identical (supplementary fig. 5, Supplementary Material online), ruling out a bias in our diversity analyses caused by differences in recombination rates between edited and unedited sites.

We also computed the iHS (Voight et al. 2006) using the software rehh (Gautier and Vitalis 2012) as a second approach to test for directional selection in favor of the derived G allele in edited sites. G alleles raising rapidly due to strong selection will have less chances to accumulate new mutations around and will tend to have high levels of haplotype homozygosity extending much further than expected under a neutral model. The rationale of the iHS approach is therefore to test whether the derived G allele at an edited site tends to segregate on an unusually long haplotype of low diversity (Voight et al. 2006). Because haplotypes cannot be inferred for pool sequencing, we computed iHS only for the DGRP2 population. Negative values of iHS indicate unusually long haplotypes carrying the derived G allele compared with the ancestral A allele. Values of iHS close to zero indicate that the haplotypes carrying both the ancestral and the derived alleles are equally large and the tested SNP is likely neutral (Voight et al. 2006).

To scan for polymorphic sites under balancing selection, we used the software ballet (DeGiorgio et al. 2014). Ballet combines intraspecies polymorphism and interspecies divergence with the spatial distribution of polymorphisms and substitutions around a selected site. The signature of balancing selection is that of a local increase in diversity relative to divergence, and a skew of the site frequency spectrum toward intermediate frequencies. The method outperforms both the HKA test and the Tajima’s *D* under a diverse set of demographic assumptions, such as a population bottleneck and growth (DeGiorgio et al. 2014). We calculated an LLR for each polymorphic site implemented in the test type T1. The input files for ME and FL populations consisted of the polymorphic state inferred from the pool-sequencing data. Because ballet can only handle a maximum of 100 lines, we used a random sample of 50 isogenic DGRP2 lines (fig. 1*d*) and of 100 randomly sampled lines to carry out the LLR computation. The result obtained for 100 lines are similar to the result for 50 lines (not shown). We specified a window size of 200 sites, as little is gained by incorporating information from additional sites (DeGiorgio et al. 2014), where a site is an intraspecies polymorphism or a divergent site. Divergent sites to *D. simulans* were defined as single nucleotide substitution: that is, homologous nonpolymorphic (fixed) sites that contain different nucleotides between *D. melanogaster* and *D. simulans*. Ballet also utilizes information regarding the recombination distance between sites. We used the recombination rate data from Comeron et al. (2012) to linearly interpolate recombination distance between two consecutive sites.

### Estimation of *f*_s_ and *f*_n_

To estimate *f*_s_ and *f*_n_ in *D. melanogaster*, we first compiled all A sites from the reference genome, release r5.40, and generated a variant call file with all potential A, G polymorphisms. We used this file as input to CooVar (Vergara et al. 2012), which analyzed the effect of each A-to-G mutation in coding regions. The output files were integrated into the DGRP2, FL, and ME polymorphism database to identify the potential A, G synonymous and nonsynonymous polymorphism that are actual A, G polymorphisms.

### Gene Expression and Codon Usage Data

We download gene expression data from the GEO (acc. GSE67505). The expression data were obtained from pooled RNA-Seq data for the DGRP2 lines, as described in the original study (Huang et al. 2015). The published expression tables are given separately for male and females in fragments per kilobase of exon per million reads mapped units. To test for correlation between gene expression levels and nonrandom usage of codons (i.e., codon bias), we downloaded two measurements of codon bias (the effective number of codons or ENC and the frequency of optimal codons or FOP) from the sebida database (Gnad and Parsch 2006) and fused the DGRP2 expression data with sebida data by means of the FlyBase gene IDs. Genes containing at least one edited site were coined edited genes and unedited genes otherwise.

### Nucleotide Profiles

The nucleotide profile around edited sites were calculated as the fraction of A, C, G, and T nucleotides at each nucleotide site upstream and downstream (±10 bp and ±1,000 bp) the edited site. For the background data, we sampled *a *=* *1,657 genic A sites and *t *=* *1,549 T sites from the *D. melanogaster* genome, where *a* and *t* are the number of annotated edited sites in the direct and inverted strands, respectively, and repeated this operation 100 times to compute the fraction of each nucleotide type at each nucleotide position upstream and downstream the sampled A/T unedited sites.

## Supplementary Material

evaa046_Supplementary_Data
