# Supplementary material for: A-to-I RNA Editing Uncovers Hidden Signals of Adaptive Genome Evolution in Animals
Source: Genome Biol Evol. 2020 Mar 7;12(4):345–57. doi: 10.1093/gbe/evaa046 (PMC7186786; doi:10.1093/gbe/evaa046)
Supplement: evaa046_Supplementary_Data [file evaa046_supplementary_data.pdf]

Supplementary Information

A-to-I RNA editing uncovers hidden signals of adaptive genome evolution in animals

Niko Popitsch[1,2,§], Christian D. Huber[3,§,*], Ilana Buchumenski[4], Eli Eisenberg[5], Michael Jantsch[6,7], Arndt von Haeseler[8,9] and Miguel Gallach[9,10].

[1]Oxford NIHR Biomedical Research Center, Wellcome Trust Center for Human Genetics. University of Oxford, Roosevelt Drive, Oxford OX3 7BN, UK.
[2]Institute of Molecular Biotechnology (IMBA), Vienna BioCenter (VBC), 1030 Vienna, Austria
[3]Australian Centre for Ancient DNA, The University of Adelaide, Adelaide, SA 5005, Australia.
[4]The Mina and Everard Goodman Faculty of Life Sciences, Bar-Ilan University, Ramat Gan 52900, Israel.
[5]Raymond and Beverly Sackler School of Physics and Astronomy and Sagol School of Neuroscience, Tel Aviv University, Tel Aviv 69978, Israel.
[6]Department for Cell- and Developmental Biology, Center for Anatomy and Cell Biology. Medical University of Vienna. Schwarzspanierstrasse 17. A-1090 Vienna. Austria.
[7]Department for Medical Biochemistry. Max F. Perutz Laboratories. Medical University of Vienna. Dr. Bohr Gasse 9. A-1030 Vienna. Austria.
[8]Bioinformatics and Computational Biology, Faculty of Computer Science, University of Vienna, A-1090 Vienna, Austria.
[9]Center for Integrative Bioinformatics Vienna. Max F. Perutz Laboratories, University of Vienna and Medical University of Vienna, A-1030 Vienna, Austria.
[10]iLabSystems. C/ Alicante, 26, bajo, Castellón, Spain.

§ These authors contributed equally to this work
*Correspondence to: christian.huber@adelaide.edu.au.

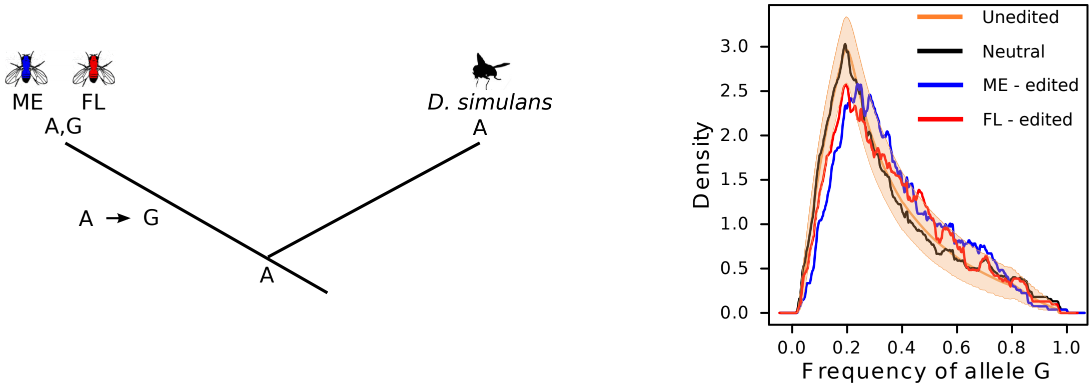

Supplementary Fig. 1. Allele frequency spectrum of the G alleles originated from A nucleotide sites in *D. melanogaster* lineage. We used *D. simulans* as an outgroup to infer the ancestral state of the A,G polymorphisms in *D. melanogaster*. Blue and red lines correspond to the frequency spectrum of the G alleles in edited sites in ME and FL populations, respectively. In black, we show the expected distribution of the G allele in edited sites if they were neutral in both populations. In peach, we show the allele frequency spectrum of the G allele in unedited sites (average and 95% confidence interval). Because the number of sequenced lines are low (39 and 86 lines for FL and ME populations, respectively), the differentiation between the genomic background and edited site is less obvious than in DGRP2 population.

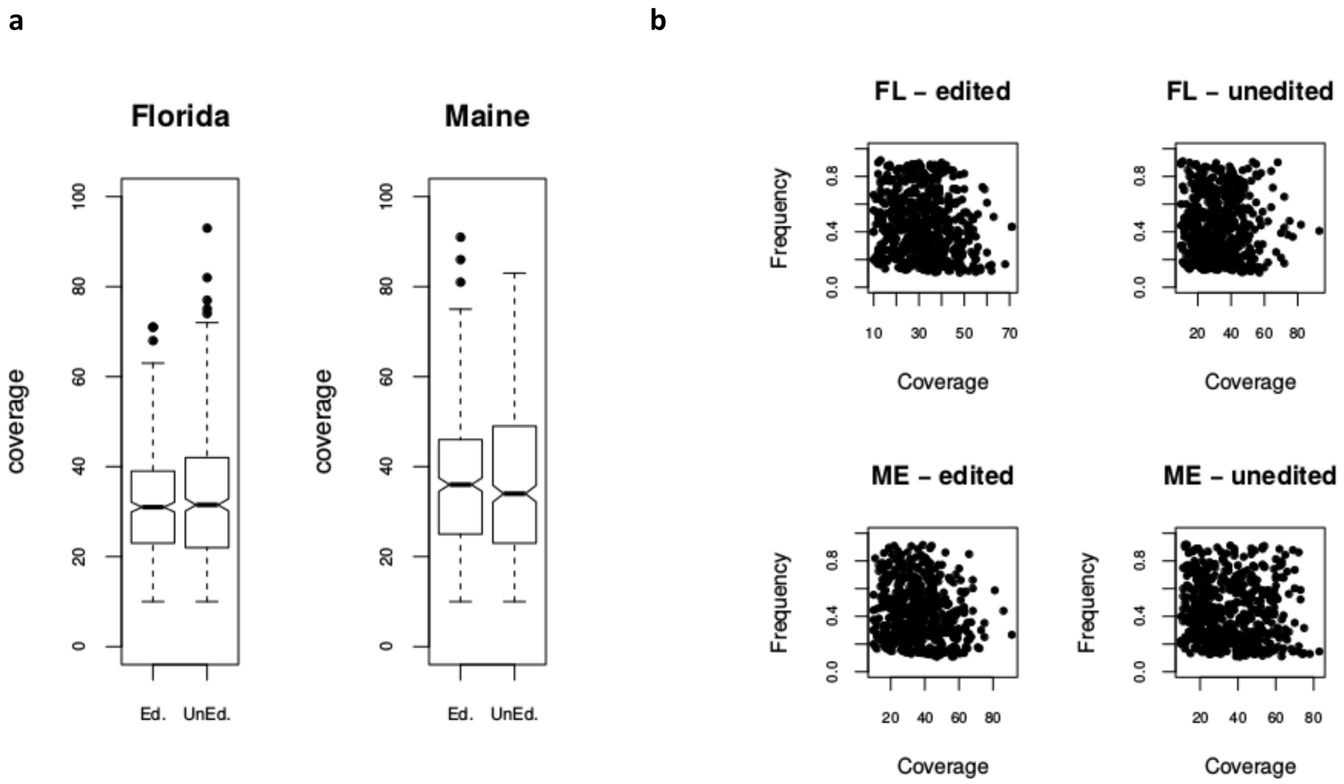

Supplementary Fig. 2. Differences in allele frequencies between edited and unedited sites is not affected by differences in sequencing coverage in *Drosophila*. a, The coverage distribution of edited sites in FL and ME populations is not different from unedited sites (*P* >> 0.05 for each paired comparison; two-sided Mann-Whitney-U test). **b,** The frequency of the minor allele and sequencing coverage at the polymorphic site do not correlate.

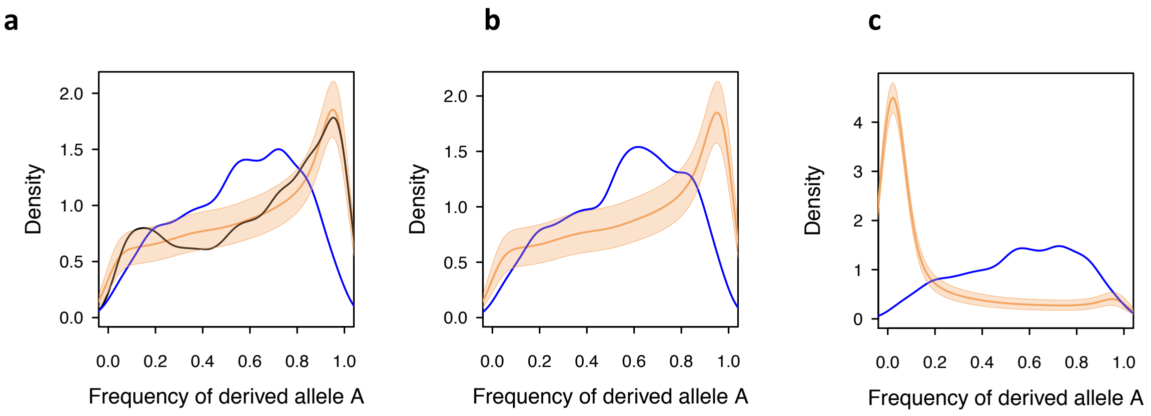

Supplementary Fig. 3. Allele frequency spectrum of the A alleles originated from G nucleotide sites in *D. melanogaster* lineage. Blue line corresponds to the frequency spectrum of the A alleles in edited sites in DGRP2 population. In black, we show the expected distribution of the A allele in edited sites if they were neutral. In peach, we show the allele frequency spectrum of the A allele in unedited sites (average and 95% confidence interval). **a,** Allele frequency spectrum of derived A alleles constrained to A sites in reference genome. It can be seen how derived A alleles segregate at lower frequencies at edited sites than at unedited sites. **b,** Same as **a**, but only for silent sites. **c,** Allele frequency spectrum of derived A alleles constrained to G sites in reference genome for genomic background and constrained to A sites in reference genome for edited sites. Comparison between **a** and **b** with **c** shows the importance of selecting sites from the same reference nucleotide type.

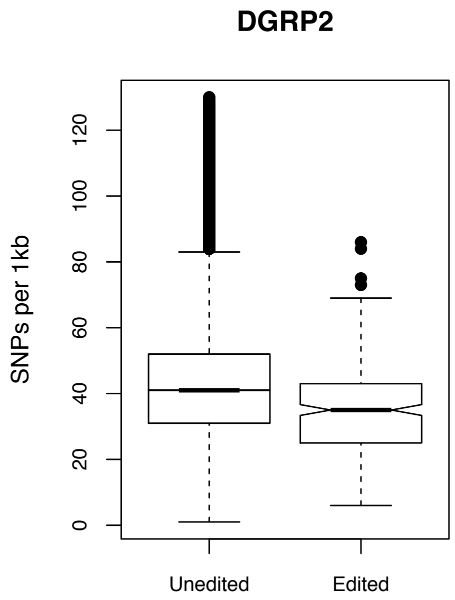

Supplementary Fig. 4. Diversity at edited and unedited sites in *Drosophila*. Windows centered on polarized A-to-G polymorphic sites have lower diversity (in SNPs per kb) for edited SNPs than for unedited SNPs (P = 8.4 x 10^{-16}; one-sided Mann-Whitney-U test).

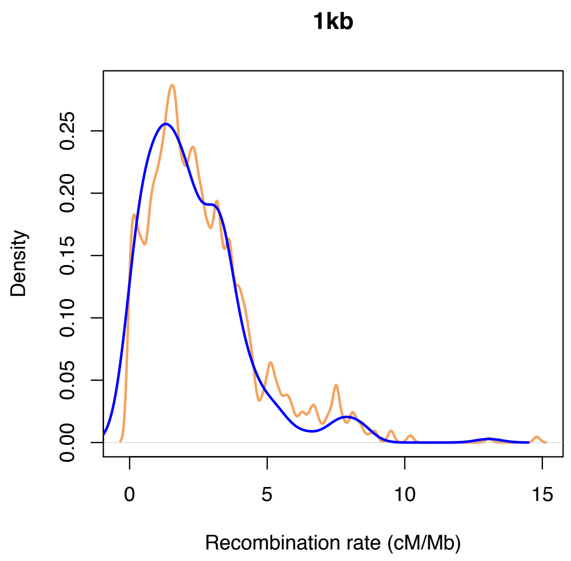

Supplementary Fig. 5. Distribution of local recombination rates at edited and unedited sites in *Drosophila*. Recombination rates were measured for windows of 1kb centered in edited (blue) and unedited (peach) sites. Both distributions are virtually identical.

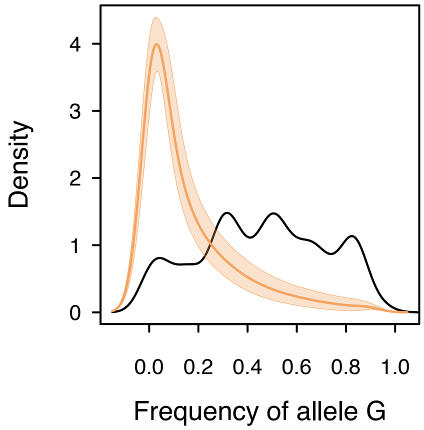

Supplementary Fig. 6. Allele frequency spectrum of intergenic G alleles originated from A nucleotide sites in human lineage. Intergenic G alleles segregate at higher frequencies in edited sites (black line) than in unedited sites (peach).

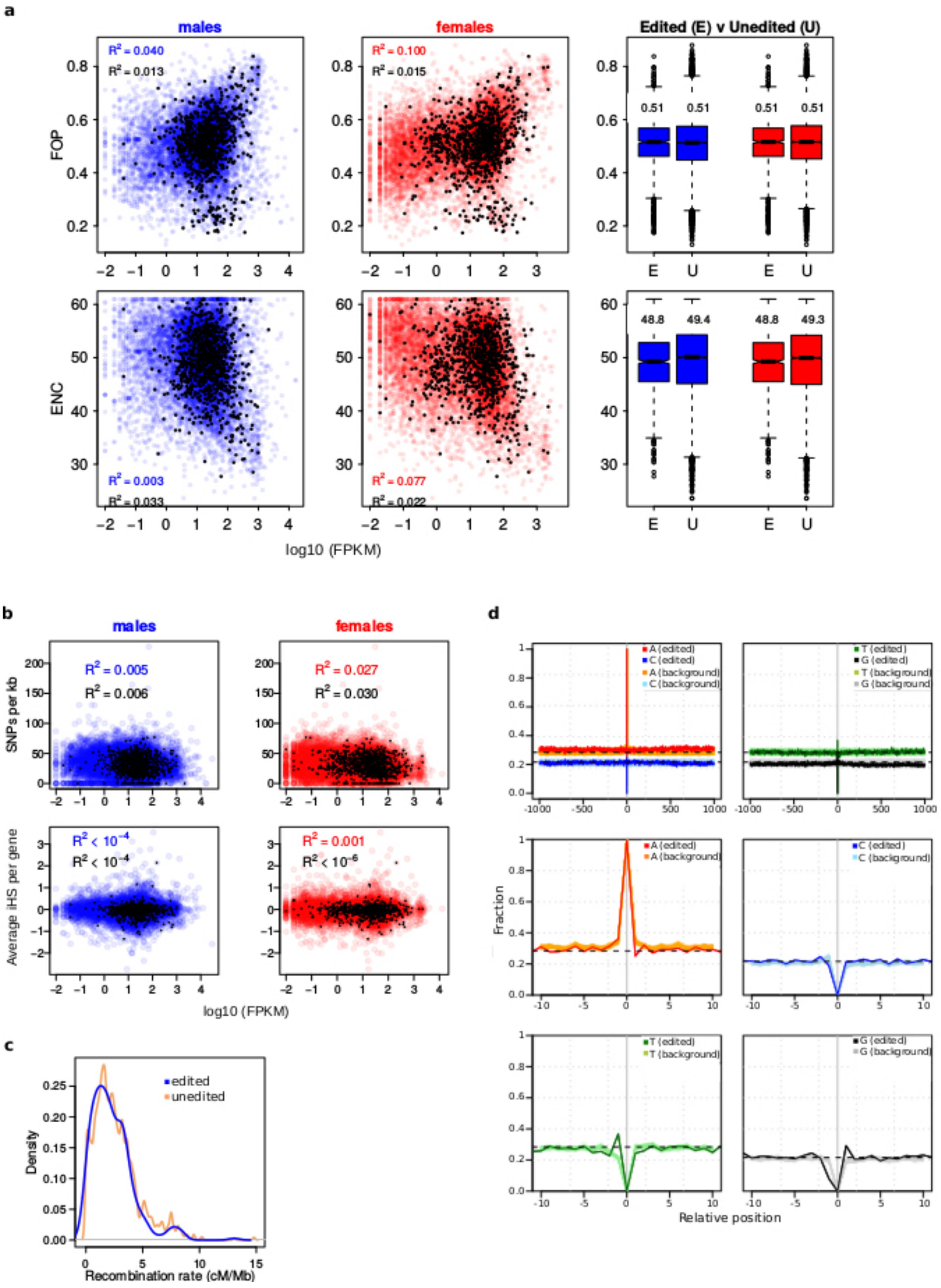
a
males
females
Edited (E) v Unedited (U)
FOP
ENC
R² = 0.040
R² = 0.013
R² = 0.100
R² = 0.015
0.51
0.51
0.51
0.51
R² = 0.003
R² = 0.033
R² = 0.077
R² = 0.022
48.8
49.4
48.8
49.3
E
U
E
U
log10 (FPKM)
b
males
females
SNPs per kb
R² = 0.005
R² = 0.006
R² = 0.027
R² = 0.030
Average iHS per gene
R² < 10⁻⁴
R² < 10⁻⁴
R² = 0.001
R² < 10⁻⁶
log10 (FPKM)
c
edited
unedited
Density
Recombination rate (cM/Mb)
d
A (edited)
C (edited)
A (background)
C (background)
T (edited)
G (edited)
T (background)
G (background)
Fraction
Relative position

Supplementary Fig. 7. Control analyses for differences in polymorphic rates and polymorphism types as a byproduct of gene expression level, recombination rate and local sequence composition in *Drosophila*. a, Bias in synonymous codon usage per gene is represented as a function of gene expression level in males (blue) and females (red). Gene expression level only explains 4% (males) to 10% (females) of the total variance in codon bias when measured as the frequency of optimal codons (FOP; the higher, the more biased) and 0.3% (males) to 7% (females) of the total variance in codon bias when measured as the effective number of codons (ENC; the lower, the more biased). The coefficient of determination for edited sites (black dots) is even lower than for unedited sites. Numbers in the boxplots refer to the mean. **b,** Nucleotide diversity (SNPs per kb per gene) and iHS (averaged per gene) does not correlate with gene expression level. Black dots: genes containing edited sites. Blue and red dots: unedited genes. **c,** Local recombination rates in 10 kb windows centered on edited (blue) and on unedited (peach) sites show identical distributions. **d,** Nucleotide profiles show that local sequence context around edited and unedited sites (±1000 bp and ±10 bp) are virtually identical.

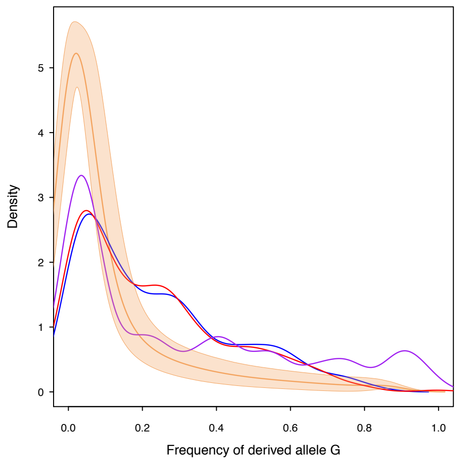

Supplementary Fig. 8. Allele frequency spectrum of the G alleles originated from A nucleotide sites in *D. melanogaster* lineage for DGRP2 population. Blue line: validated data from St. Laurent et al. [8] Red line: data from RADAR [37]. Purple line: data form Yu et al. [11] In peach, we show the allele frequency spectrum of the derived G allele in unedited sites (average and 95% confidence interval).

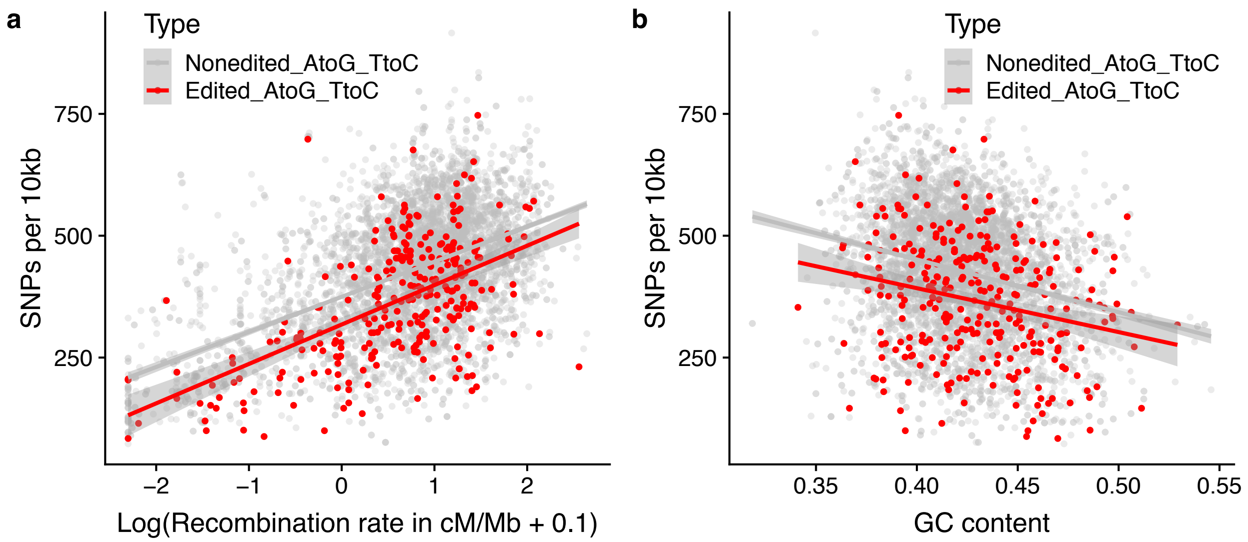

Supplementary Fig. 9. Number of SNPs in 10 kb windows as a function of (a) recombination rate and (b) GC content. The 10 kb windows are centered on A-to-G mutations that are either edited (red) or unedited (grey). Note that even though SNP number is a function of both recombination rate and GC content, there is an effect of editing reducing the SNP number that cannot be explained by these two factors. Linear regression lines are fitted to the data.

Supplementary Table 1. Number of A,G polymorphisms (and percentages) in coding regions of different *D. melanogaster* populations.

	Edited			Unedited		
	**DGRP2**	**ME**	**FL**	**DGRP2**	**ME**	**FL**
**Polymorphic A,G sites**	319 (31%)	218 (22%)	227 (22%)	113,973 (2%)	25,547 (0.5%)	27,250 (0.5%)
**Total A sites**		1,015			5,225,594	

Supplementary Table 2. Number of polarized polymorphisms in the genome of three *Drosophila* populations.

	DGRP 2		Florida		Maine	
	**Total**	**A-to-G**	**Total**	**A-to-G**	**Total**	**A-to-G**
**Edited**	755	303	543	179	507	155
**Unedited**	3,951,070	462,498	1,367,160	125,628	1,235,454	110,689

Supplementary Table 3. Number of single nucleotide polymorphism sites and polymorphism types among edited sites in DGRP2.

	Edited sites		
	**St. Laurent et al. validated**	**RADAR**	**Yu et al.**
**Polymorphic**	**372 (17%)**	**805 (16%)**	**163 (13%)**
**Not polymorphic**	1,825 (83%)	4,220 (84%)	1,111 (87%)
**Polymorphism A,G**	**349 (94%)**	**780 (97%)**	**159 (97%)**
**A,C**	10 (3%)	7 (1%)	3 (2%)
**A,T**	13 (3%)	18 (2%)	1 (1%)

St. Laurent et al. validated: Ref. 8.
RADAR: Ref. 37.
Yu et al.: Ref. 11.
In bold: increased proportion in edited sites compared to unedited sites.

Supplementary Table 4. Potential A,G replacements at non-coding and intergenic regions in *Drosophila* populations.

Population	Potential A,G replacements at non-coding and intergenic regions				
	Edited (l^{edited} = 2,544)		Genome (l = 29,325,288)		Ratio
	Polymorphic	Rate (f_l^{edited})	Polymorphic	Rate (f_l)	f_l^{edited} / f_l
DGRP2	411	0.162	702,149	0.024	6.750
ME	281	0.110	1,180,129	0.040	2.750
FL	310	0.122	1,065,262	0.036	3.389

Supplementary Table 5. Testing for an effect of editing on the polymorphism to divergence ratio in 10kb surrounding window, and on iHS, using the DGRP2 data.

**Polymorphism to divergence ratio in 10kb windows centered on A-to-G**				
Filter on G allele frequency	Factor	Estimate	Std. Error	p-value
No filter	Intercept	0.538577	0.04198	< 2e-16 ***
	log(Recombination rate + 0.1)	0.127586	0.003301	< 2e-16 ***
	GC content	0.232169	0.098863	0.01889 *
	Editing status (edited)	-0.040343	0.012631	0.00141 **
> 5%	Intercept	0.505884	0.06575	2.15e-14 ***
	log(Recombination rate + 0.1)	0.122606	0.005303	< 2e-16 ***
	GC content	0.339607	0.154933	0.028488 *
	Editing status (edited)	-0.051553	0.014925	0.000563 ***
> 10%	Intercept	0.436508	0.07661	1.44e-08 ***
	log(Recombination rate + 0.1)	0.110643	0.006339	< 2e-16 ***
	GC content	0.513387	0.180688	0.004550 **
	Editing status (edited)	-0.057072	0.01673	0.000663 ***
**iHS of A-to-G polymorphisms**				
Filter on G allele frequency	Factor	Estimate	Std. Error	p-value
No filter	Intercept	0.60326	0.19705	0.00221 **
	log(Recombination rate + 0.1)	0.02242	0.01643	0.17253
	GC content	1.46664	0.46186	0.00150 **
	Editing status (edited)	-0.13301	0.06869	0.05286 .
> 10%	Intercept	0.42848	0.21843	0.0499 *
	log(Recombination rate + 0.1)	-0.01398	0.01855	0.451
	GC content	-0.98617	0.51237	0.0543
	Editing status (edited)	-0.14924	0.07467	0.0457 *
